# Quorum-sensing non-coding small RNAs use unique pairing regions to differentially control mRNA targets

**DOI:** 10.1111/j.1365-2958.2011.07959.x

**Published:** 2012-01-09

**Authors:** Yi Shao, Bonnie L Bassler

**Affiliations:** 1 Department of Molecular Biology, Princeton UniversityPrinceton, NJ, USA; 2 Howard Hughes Medical InstituteChevy Chase, MD, USA

## Abstract

Quorum sensing is a mechanism of cell–cell communication that bacteria use to control collective behaviours including bioluminescence, biofilm formation and virulence factor production. In the *Vibrio harveyi* and *Vibrio cholerae* quorum-sensing circuits, multiple non-coding small regulatory RNAs called the quorum-regulated small RNAs (Qrr sRNAs) function to establish the global quorum-sensing gene expression pattern by modulating translation of multiple mRNAs encoding quorum-sensing regulatory factors. Here we show that the Qrr sRNAs post-transcriptionally activate production of the low cell density master regulator AphA through base pairing to *aphA* mRNA, and this is crucial for the accumulation of appropriate levels of AphA protein at low cell density. We find that the Qrr sRNAs use unique pairing regions to discriminate between their different targets. Qrr1 is not as effective as Qrr2–5 in activating *aphA* because Qrr1 lacks one of two required pairing regions. However, Qrr1 is equally effective as the other Qrr sRNAs at controlling targets like *luxR* and *luxO* because it harbours all of the required pairing regions for these targets. Sequence comparisons reveal that *Vibrionaceae* species possessing only *qrr*1 do not have the *aphA* gene under Qrr sRNA control. Our findings suggest co-evolving relationships between particular Qrr sRNAs and particular mRNA targets.

## Introduction

Quorum sensing is the chemical communication process bacteria use to regulate gene expression in response to changes in cell population density. Quorum sensing relies on the production, secretion and subsequent detection of extracellular signalling molecules called autoinducers (AIs). Quorum sensing ensures that bacteria behave as individuals at low cell density and exhibit group behaviours at high cell density. Quorum-sensing-controlled behaviours include bioluminescence, biofilm formation and virulence factor production (Davies *et al*., 1998; Zhu *et al*., 2002; Hammer and Bassler, 2003; Ng and Bassler, 2009). Multiple non-coding small regulatory RNAs lie at the centres of the *Vibrio harveyi* and *Vibrio cholerae* quorum-sensing circuits and are the focus of this study (Lenz *et al*., 2004; Tu and Bassler, 2007).

Non-coding small RNAs (sRNAs) are widely used regulators in bacteria and eukaryotes. In bacteria, they control traits including nutrient uptake, stress response, viral immunity, and in the present context, quorum sensing (Waters and Storz, 2009). Bacterial sRNAs are classified according to their regulatory mechanism. There are protein activity modulating sRNAs, *cis*-encoded base pairing sRNAs, *trans*-encoded base pairing sRNAs, and the recently discovered CRISPR sRNAs (Waters and Storz, 2009). The quorum-regulated sRNAs called the Qrr sRNAs in the *V. harveyi* and *V. cholerae* quorum-sensing systems belong to the set of *trans*-acting sRNAs that function through Hfq-assisted base pairing with target mRNAs to control mRNA translation or stability (Caron *et al*., 2010). This class of sRNAs can repress mRNA translation by pairing with the ribosome binding site and occluding ribosome access, typically resulting in mRNA degradation (Aiba, 2007). Alternative mechanisms exist in which sRNAs pair within mRNA coding regions or in intergenic regions of polycistronic transcripts, which leads to RNase E- or RNase III-dependent endonucleolytic cleavage (Desnoyers *et al*., 2009; Pfeiffer *et al*., 2009; Papenfort *et al*., 2010). sRNAs can also act as activators by pairing with and altering the secondary structures of regions in the 5′ UTR of mRNAs to reveal ribosome binding sites, typically promoting mRNA stabilization and translation (Frohlich and Vogel, 2009). Activation can also occur through sRNA generation of accessible ribosome binding sites via endonucleolytic cleavage or formation of a nuclease barrier at the 5′ end of the target mRNA (Obana *et al*., 2010; Ramirez-Pena *et al*., 2010).

**Fig 1:**
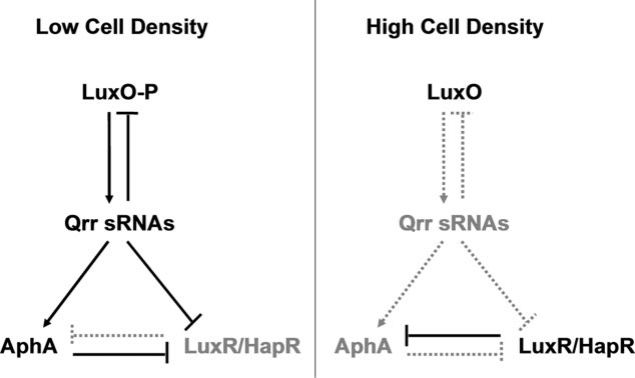
Model for Qrr sRNA regulation of *aphA*, *luxR*/*hapR* and *luxO*. At low cell density, phospho-LuxO activates expression of the *qrr* genes encoding the Qrr sRNAs. The Qrr sRNAs promote translation of the low cell density master regulator AphA and inhibit translation of the high cell density master regulator LuxR/HapR. At high cell density, Qrr sRNA production ceases because dephosphorylated LuxO is inactive. AphA translation stops and LuxR/HapR translation occurs. LuxO production is repressed by the Qrr sRNAs in a negative feedback loop. AphA and LuxR repress each other at the transcriptional level.

In *V. harveyi* quorum sensing, at low cell density, in the absence of AIs, the quorum-sensing response regulator protein LuxO is phosphorylated (Freeman and Bassler, 1999). Phospho-LuxO activates the expression of five genes (*qrr*1–5) encoding the five Qrr sRNAs (Tu and Bassler, 2007). The Qrr sRNAs activate translation of the low cell density master regulator AphA, which controls ∼ 300 low cell density target genes (Rutherford *et al*., 2011). The Qrr sRNAs simultaneously repress translation of the high cell density master regulator LuxR (Fig. 1, left) (Tu and Bassler, 2007). At high cell density, when AIs are present, LuxO is dephosphorylated and it is inactive, so production of the Qrr sRNAs ceases. In the absence of the Qrr sRNAs, AphA is not produced, but LuxR translation occurs. LuxR controls ∼ 700 high cell density target genes (Fig. 1, right) (J.C. van Kessel, unpublished). The quorum-sensing circuit of the closely related pathogenic bacterium *V. cholerae* resembles that of *V. harveyi*, but *V. cholerae* only has Qrr1–4 and the *V. cholerae* LuxR homologue is called HapR (Lenz *et al*., 2004). In *V. harveyi* and *V. cholerae*, in addition to controlling the two quorum-sensing master regulators, AphA and LuxR/HapR, the Qrr sRNAs control other targets and they participate in several feedback loops. These Qrr sRNA-mediated feedback loops fine-tune the quorum-sensing output by providing robust responses to cell population density changes, promoting high fidelity signal transmission, and controlling the input–output dynamic range (Svenningsen *et al*., 2008; 2009; Tu *et al*., 2008; 2010; Ng and Bassler, 2009; Teng *et al*., 2011).

In this study, we characterize the production pattern of the newly identified quorum-sensing low cell density master regulator AphA in both *V. harveyi* and *V. cholerae*. We show that the Qrr sRNAs activate AphA production through direct base pairing to the *aphA* mRNA 5′ UTR, and this regulatory step is crucial for proper AphA protein accumulation at low cell density. We also find that the Qrr sRNAs use a unique set of pairing regions to activate *aphA* compared with the regions they use to control other target mRNAs such as *luxR* and *luxO*. Qrr1 is less effective than the other Qrr sRNAs in activating *aphA* because it lacks one of the critical pairing regions. However, Qrr1 is fully functional in its control of mRNA targets that do not require this particular pairing region. Sequence analysis reveals that *Vibrionaceae* species can possess 1, 4 or 5 Qrr sRNAs. Our evidence indicates that the Qrr-*aphA* mRNA interaction does not occur in *Vibrionaceae* species possessing only Qrr1. Rather, only vibrios containing multiple Qrr sRNAs control *aphA* by this mechanism. We propose that harbouring multiple Qrr sRNAs enables the Qrr sRNAs to diversify and evolve distinct target preferences, and in this case, to ensure optimized quorum-sensing gene expression (Tu *et al*., 2008).

## Results

### AphA production is repressed at high cell density

In *V. harveyi* and *V. cholerae*, *aphA* mRNA levels decrease when cells enter high cell density mode. This reduction occurs because LuxR/HapR (which is produced at high cell density) represses *aphA* transcription, and the absence of the Qrr sRNAs (which are made at low cell density) decreases *aphA* mRNA stabilit*y* (Rutherford *et al*., 2011). To understand how this regulation affects AphA protein levels, we measured AphA protein by Western blot in four different *V. harveyi* and *V. cholerae* genetic backgrounds: wild type (high cell density mode), *luxO*D47E (mimicking phospho-LuxO, low cell density mode), *ΔluxR/ΔhapR* (high cell density mode, but LuxR/HapR independent), *luxO*D47E *ΔluxR/ΔhapR* (low cell density mode, but LuxR/HapR independent).

**Fig 2:**
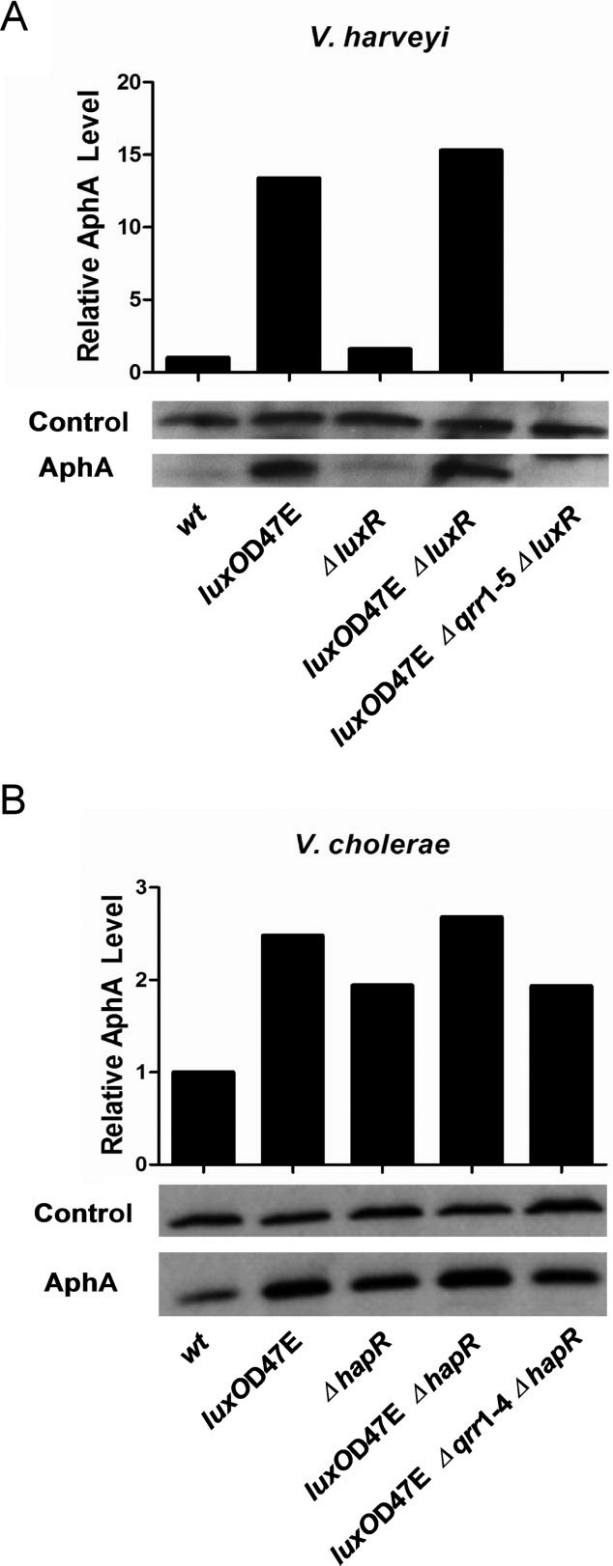
AphA production is repressed at high cell density. A. AphA protein levels in *V. harveyi* wild type (BB120), *luxO*D47E (KM83), *ΔluxR* (KM669), *luxO*D47E *ΔluxR* (KM812), and *luxO*D47E *Δqrr*1–5 *ΔluxR* (YS040). B. AphA protein levels in *V. cholerae* wild type (C6706), *luxO*D47E (SLS340), *ΔhapR* (SLS390), *luxO*D47E *ΔhapR* (SLS640), and *luxO*D47E *Δqrr*1–4 *ΔhapR* (SLS641). Cells were harvested at OD_600_ ∼ 1.0, and protein levels were determined using Western blot.

We begin with the *V. harveyi* results: compared with when cells are in low cell density mode, AphA protein is dramatically reduced when *V. harveyi* is in high cell density mode (Fig. 2A, compare wild type with *luxO*D47E), which is consistent with AphA having its primary function as the low cell density master regulator (Rutherford *et al*., 2011). Analogous results were obtained in the *V. harveyi luxR* deletion strains (Fig. 2A, compare *ΔluxR* with *luxO*D47E *ΔluxR*). The *luxO*D47E *Δqrr*1–5 *ΔluxR* strain shows that it is indeed the Qrr sRNAs that are responsible for inducing the high-level production of AphA observed at low cell density (Fig. 2A). Again, these results are consistent with our previous genetic finding that, at low cell density, the Qrr sRNAs activate *aphA* translation independently of LuxR (Rutherford *et al*., 2011). AphA protein levels are slightly higher in the *V. harveyi ΔluxR* strain compared with the *V. harveyi* wild type, and in the *V. harveyi luxO*D47E *ΔluxR* double mutant compared with the *V. harveyi luxO*D47E single mutant, which is consistent with the fact that LuxR represses transcription of *aphA* (Fig. 2A) (Pompeani *et al*., 2008; Rutherford *et al*., 2011). Taken together these results show that *V. harveyi* strains in high cell density mode have significantly less AphA protein than do *V. harveyi* strains in low cell density mode. Furthermore, the relative differences in AphA protein levels in the various strains show that while LuxR negatively regulates *aphA* at the transcriptional level, Qrr sRNA-mediated post-transcriptional activation plays a much larger regulatory role.

In the companion set of experiments examining *V. cholerae*, we find that the AphA protein exhibits a similar pattern to that of *V. harveyi*: AphA is lower in high cell density mode cells than in low cell density mode cells, in both the presence and absence of HapR (Fig. 2B). There is one dramatic difference between the *V. harveyi* and *V. cholerae* results. In *V. cholerae*, unlike in *V. harveyi*, at high cell density there remains detectable AphA protein. Thus, in *V. harveyi* it appears that Qrr sRNA activation of *aphA* is all or none. In *V. cholerae*, by contrast, the Qrr sRNAs appear to fine-tune AphA levels.

### AphA production is activated by the Qrr sRNAs through base pairing

**Fig 3:**
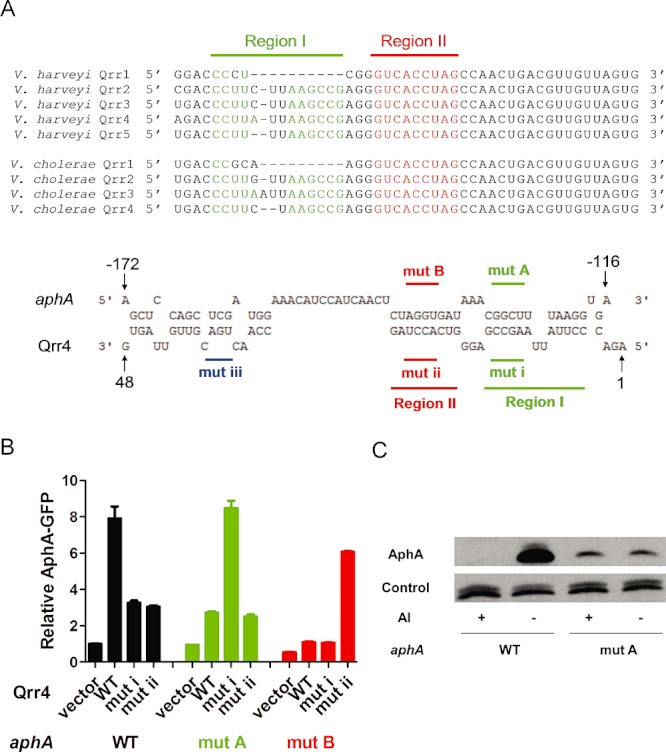
Qrr sRNAs activate *aphA* expression through direct base pairing. A. RNA sequence alignment of the five *V. harveyi* Qrr sRNAs and the four *V. cholerae* Qrr sRNAs; the 5′ end highly conserved 1–48 nt is shown (Lenz *et al*., 2004; Tu and Bassler, 2007). Pairing regions between *aphA* mRNA and the multiple Qrr sRNAs are highlighted as region I and region II, coloured in green and red respectively. RNA sequence alignment of *V. harveyi* Qrr4 (5′ end 1–48 nt) with *aphA* mRNA 5′ UTR by the online freely available RNAhybrid software (http://bibiserv.techfak.uni-bielefeld.de/rnahybrid/submission.html). The sequences of the *qrr*4 mutation in pYS121 (mutation i, denoted mut i) and in pYS120 (mutation ii, denoted mut ii) are indicated below, and the sequences of the corresponding AphA-GFP mutation in pYS113 (mutation A, denoted mut A) and pYS112 (mutation B, denoted mut B) are indicated above. B. Fluorescence from plasmid-encoded *V. harveyi* AphA-GFP (pYS069) or mutant AphA-GFP (pYS113 or pYS112) translational fusions were measured in *E. coli* MC4100 carrying an empty vector (pRHA109), a vector expressing a rhamnose-inducible *qrr*4 gene (pSTR0227) or a mutant *qrr*4 gene (pYS121 or pYS120). GFP from three independent cultures was measured for each strain and the means and SEMs are shown. C. AphA protein levels in a *V. harveyi ΔluxM ΔluxPQ ΔcqsS ΔluxR* strain with wild-type *aphA* (YS010) or *aphA* carrying mutation A (see panel A) (YS034), with or without exogenous 10 µM autoinducer (AI; 3OHC4 homoserine lactone). Cells were harvested at OD_600_ ∼ 1.0, and protein levels were determined using Western blot.

The Qrr sRNAs belong to the family of Hfq-dependent *trans*-acting sRNAs which act by base pairing to their target mRNAs (Lenz *et al*., 2004; Tu and Bassler, 2007). Furthermore, we know that the Qrr sRNAs regulate other targets by direct base pairing (Hammer and Bassler, 2007; Svenningsen *et al*., 2009; Tu *et al*., 2010; Bardill *et al*., 2011; Teng *et al*., 2011). We wondered if this is the case for Qrr sRNA activation of *aphA*. Sequence comparison of the *V. harveyi* and *V. cholerae aphA* mRNAs with the Qrr sRNAs reveals a potential Qrr binding site located ∼ 130 nt upstream of the start codon in the 5′ UTR of the *aphA* mRNA. The complementary sequence in the Qrr sRNAs is comprised of two sections, which we name region I and region II (Fig. 3A). The extensive complementarity suggests that the Qrr sRNAs could control AphA production through base pairing between one or both of these regions. We again begin with *V. harveyi* to test this idea. First, a plasmid encoding a *V. harveyi* AphA–GFP translational fusion driven by an IPTG inducible promoter (pYS069) was engineered into *Escherichia coli*. *E. coli* was used to avoid interference from other *V. harveyi* quorum-sensing components that could alter *aphA* regulation. Second, we introduced a plasmid encoding *V. harveyi* Qrr4 under a rhamnose-inducible promoter (pSTR0227) into the *E. coli* strain carrying the AphA–GFP fusion. We chose Qrr4 as a representative of the set of the Qrr sRNAs. AphA-GFP production increased when wild-type *V. harveyi* Qrr4 was expressed in *E. coli* (Fig. 3B, columns 1 and 2), showing that the Qrr sRNAs act independently of other vibrio factors to activate AphA protein production, which suggests a base pairing mechanism.

To examine the requirements for base pairing, we introduced mutations into the *qrr*4 gene in the sequences predicted to pair with the *aphA* mRNA. We engineered mutations into each of the two predicted pairing regions (Fig. 3A). The mutations are an AGCC to UCGG alteration in region I of Qrr4 and an ACCU to UGGA change in region II of Qrr4, which we call mutation i and mutation ii respectively. Both Qrr4 mutation i and mutation ii eliminated activation of AphA-GFP production, demonstrating that the sequences in these regions of Qrr4 are crucial for activation (Fig. 3B, columns 3 and 4). We obtained similar results when the corresponding mutations in these predicted pairing regions were introduced into the 5′ UTR of the AphA-GFP reporter (Fig. 3A). In this case, we mutated GGCU to CCGA to disrupt pairing to Qrr4 region I and we altered AGGU to UCCA to disrupt pairing to Qrr4 region II. We call these constructs mutation A and mutation B respectively. Figure 3B shows that introduction of mutation A or mutation B prevented full *aphA* activation by wild-type Qrr4, again suggesting that the two predicted regions are important for regulation (Fig. 3B, columns 5 and 6 and 9 and 10). Finally, introduction of each complementary pair of mutations into Qrr4 (mutation i or mutation ii) and AphA-GFP (mutation A or mutation B) to restore base pairing led to full activation. By contrast, combining non-complementary mutations did not restore regulation. That is, mutation ii in Qrr4 could not fully activate mutation A in *aphA*, and likewise mutation i in Qrr4 could not fully activate mutation B in *aphA* (Fig. 3B, columns 7 and 8 and 11 and 12). Taken together, these findings show that Qrr4 activates AphA production at low cell density through base pairing to the *aphA* mRNA 5′ UTR. Furthermore, both region I and region II of Qrr4 are required for full function. Exactly analogous results were obtained for *V. cholerae* AphA-GFP (Fig. S1A). We note that there is a significant difference in basal levels of AphA in *V. harveyi* and *V. cholerae* (Fig. 2). We do not observe such dramatic differences in protein production from the fusion constructs (Figs 3B and S1A). Possibly, additional mRNA sequences that are not included in our AphA-GFP clones influence protein production. Alternatively, other *V. cholerae* factors could exist that influence AphA production. Quantitative RT-PCR experiments using multiple primer pairs covering the *aphA* 5′ UTR and coding sequence indicate that no *aphA* processing occurs during activation (data not shown). Finally, and not surprisingly, Hfq is required for productive Qrr-*aphA* mRNA interactions as no Qrr activation of *aphA* occurred in a Δ*hfq E. coli* strain (data not shown).

To show that proper base pairing between the Qrr sRNAs and the *aphA* mRNA affects AphA protein production, we introduced mutation A into the 5′ UTR of the *aphA* gene and crossed it into the *V. harveyi* chromosome. To control whether the cells were in low cell density or high cell density mode, we engineered this mutation into a *V. harveyi* mutant that only performs quorum sensing in response to exogenously supplied AI. Thus, in the absence of AI, this strain is locked in low cell density mode, whereas, in the presence of AI the strain is locked in high cell density mode. We used Western blot to monitor AphA protein. In the strain with wild-type *aphA*, AphA protein level decreased dramatically following addition of a saturating amount of AI. By contrast, AphA protein was low in the presence and absence of AI in the base pairing deficient mutant (Fig. 3C). We conclude that Qrr sRNA activation is important for appropriate AphA protein accumulation at low cell density in *V. harveyi*. Similar results were obtained in *V. cholerae* (Fig. S1B)

### The Qrr sRNAs use distinct pairing regions to control different targets

**Fig 4:**
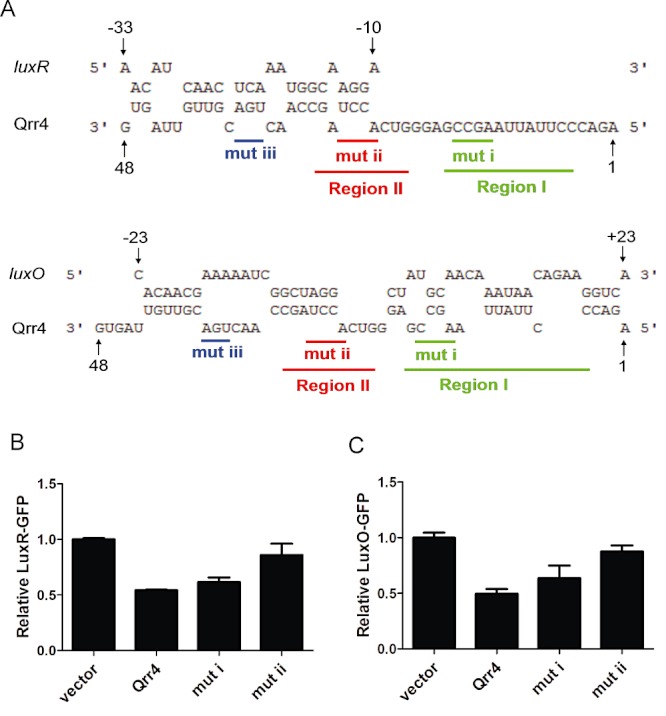
Qrr sRNAs use unique pairing regions to activate *aphA* expression. A. RNA sequence alignment of the *V. harveyi luxR, luxO* mRNA with *V. harveyi* Qrr4 (5′ end 1–48 nt) by RNAhybrid as in Fig. 3A. Pairing region I and region II are coloured in green and red respectively. The *qrr*4 mutation in pYS121 (mutation I, denoted mut i) and pYS120 (mutation ii, denoted mut ii) are indicated below the sequences. B and C. Fluorescence from plasmid-encoded *V. harveyi* LuxR-GFP (pYS141) and LuxO-GFP (pYS142) translational fusions were measured in *E. coli* MC4100 carrying an empty vector (pRHA109), a vector expressing a rhamnose-inducible *qrr*4 gene (pSTR0227), or a mutant *qrr*4 gene (pYS120 or pYS121). GFP from three independent cultures was measured for each strain and the means and SEMs are shown.

The Qrr sRNAs regulate multiple targets both in *V. harveyi* and *V. cholerae* in addition to the three central quorum-sensing components *aphA*, *luxR/hapR* and *luxO* (Lenz *et al*., 2004; Hammer and Bassler, 2007; Tu and Bassler, 2007; Svenningsen *et al*., 2009; Tu *et al*., 2010; Bardill *et al*., 2011; Rutherford *et al*., 2011; Teng *et al*., 2011). Given that a variety of targets must be regulated properly *in vivo*, we wondered whether the Qrr sRNAs can discriminate between target mRNAs (Figs 3A, 4A and S2).

To test this idea, we chose to study *aphA*, *luxR* and *luxO* because these three targets are common to both *V. harveyi* and *V. cholerae*. The sequence alignments shown in Figs 3A and 4A indicate that only one of the two regions we identified as important for *aphA* regulation (region II) is complementary to *luxR* and *luxO* mRNA. Because no other potential Qrr4 pairing sequences could be identified in the *luxR* and *luxO* mRNA, these findings suggest a mechanism for how differential Qrr sRNA regulation could be achieved. Specifically, particular regions of the Qrr sRNAs could be employed for regulation of different target mRNAs. To test this possibility, we again used *E. coli*, this time containing an AphA–GFP, LuxR–GFP (pYS141) or LuxO–GFP (pYS142) translational fusion. GFP production levels were measured in each case in the presence of wild-type Qrr4 and the Qrr4 mutants described above harbouring alterations in region I or region II. As expected, wild-type Qrr4 activated AphA-GFP and repressed LuxR-GFP and LuxO-GFP (first two columns in Figs 3B, 4B and C). Also as shown above, mutations in either region I or region II of Qrr4 compromised activation of AphA-GFP (Fig. 3B, columns 3 and 4). By contrast, only the mutation in region II affected repression of LuxR-GFP and LuxO-GFP (final two columns in Fig. 4B and C). Thus, we conclude that the Qrr sRNAs employ distinct pairing regions to discriminate between different targets, and for the three targets we tested, the region we call region I is uniquely used for *aphA* activation, while region II is used for all three targets (see also *Discussion* and Fig. S5).

### Sequence analyses of vibrio *qrr* and *aphA* genes suggest co-evolution

**Fig 5:**
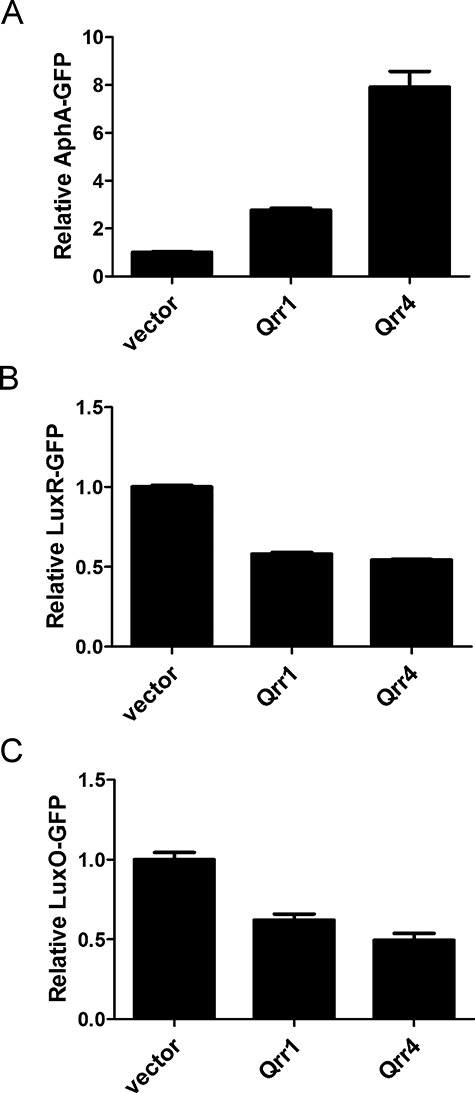
Qrr1 shows different capabilities in regulating multiple targets. Fluorescence from plasmid-encoded *V. harveyi* AphA-GFP [pYS069; (A)], LuxR-GFP [pYS141; (B)], LuxO-GFP [pYS142; (C)] translational fusions were measured in *E. coli* MC4100 carrying an empty vector (pRHA109), a vector expressing a rhamnose-inducible *qrr*1 gene (pYS122) or *qrr*4 gene (pSTR0227). GFP from three independent cultures was measured for each strain and the means and SEMs are shown.

The region II sequence employed by the Qrr sRNAs to pair with *aphA*, *luxR* and *luxO* mRNA is conserved among all the Qrr sRNAs: five Qrr sRNAs in *V. harveyi* and four Qrr sRNAs in *V. cholerae*. However, while *V. harveyi* and *V. cholerae* Qrr1 contains region II, it lacks region I, which we found is critical for pairing with *aphA* mRNA (Fig. 3A). We would therefore surmise that Qrr1 should be less effective in regulating *aphA* than are the other Qrr sRNAs. Furthermore, our above experiments predict that region I is not involved in pairing with *luxR* and *luxO* mRNA, thus we further suspect that Qrr1 should work as effectively as Qrr4 in regulating *luxR* and *luxO*. To test these predictions, we compared the strength of Qrr4 and Qrr1 regulation of *aphA*, *luxR* and *luxO* using the GFP reporters in *E. coli*. Indeed, Qrr1 is roughly threefold less effective at activating AphA-GFP than is Qrr4 (Fig. 5A). However, both Qrr1 and Qrr4 repress LuxR-GFP and LuxO-GFP production to similar levels (Fig. 5B and C).

**Fig 6:**
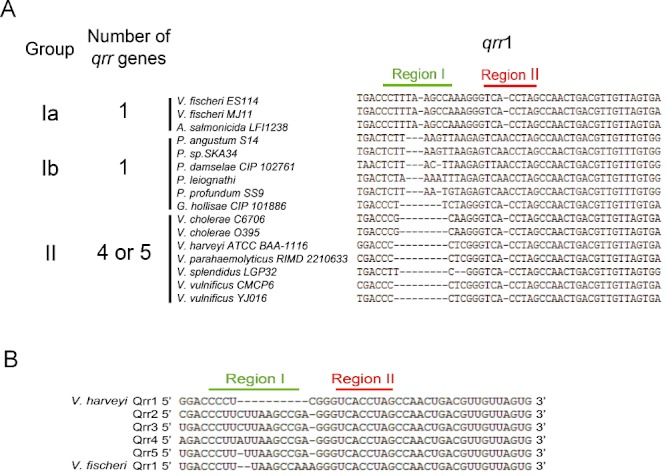
Features of Qrr sRNAs-*aphA* pairing regions in different vibrios. A. Sequence alignment of *qrr*1 genes in different *Vibrionaceae* species assigned to three groups (Ia) no *aphA* gene (Ib) *aphA* presumably not under Qrr sRNA control (II) *aphA* under Qrr sRNA control. The number of *qrr* genes in each species is shown on the left. B. RNA sequence alignment of Qrr1–5 in *V. harveyi* with Qrr1 of *V. fischeri*. The *aphA* pairing region I and region II are indicated above the sequences as in Fig. 3A.

The fact that Qrr1 lacks region I becomes more interesting when we examine the genome sequences of other species in the *Vibrionaceae* family. All sequenced *Vibrionaceae* species can be placed into two major groups: those species containing only the *qrr*1 gene located next to the gene encoding the quorum-sensing response regulator protein LuxO (for example, *Vibrio fischeri*) and those species harbouring either four or five *qrr* genes including *qrr*1 (for example *V. cholerae* and *V. harveyi*) (Fig. 6A) (Miyashiro *et al*., 2010). The species that possess multiple *qrr* genes also possess highly conserved *aphA* genes. (Fig. 6A group II, Fig. S3). In each of these *aphA* mRNA 5′ UTRs, the sequences required to pair with the Qrr regions I and II are also highly conserved (Fig. 6A group II, Fig. S4). However, in *Vibrionaceae* species containing only *qrr*1, either no *aphA* gene exists (for example, *V. fischeri*, Fig. 6A group Ia) or a less well-conserved *aphA*-type gene exists and it lacks the entire Qrr pairing region in the 5′ UTR (for example, *Photobacterium angustum*, Fig. 6A group Ib). Finally, in the species containing only *qrr*1, *qrr*1 is more similar to *qrr*2–5 in *V. harveyi* and *qrr*2–4 in *V. cholerae*, than it is to the *qrr*1 genes of *V. harveyi* and *V. cholerae* (Figs 3A and 6B, *V. fischeri* is shown as the example). Taken together, our results suggest that evolution of multiple *qrr* genes in vibrios is linked to newly emerged targets that are under their control. Presumably, in *V. cholerae* and *V. harveyi* following duplication of the ancestral *qrr*1 gene, Qrr1 became dedicated to regulation of targets including *luxR* and *luxO*, while the other Qrr sRNAs became available to control additional targets, such as *aphA*.

## Discussion

A set of highly conserved Qrr sRNAs function at the core of the *V. harveyi* and *V. cholerae* quorum-sensing circuits. The Qrr sRNAs are expressed when cells are in low cell density mode and they act to repress the production of the high cell density master regulator LuxR/HapR (Lenz *et al*., 2004; Tu and Bassler, 2007; Bardill *et al*., 2011). Recently, the Qrr sRNAs were also shown to activate the production of the low cell density master regulator AphA (Rutherford *et al*., 2011). As AphA and LuxR/HapR control hundreds of target genes at low cell density and high cell density, respectively, and they mutually repress each other at the transcriptional level, the amount of the Qrr sRNAs present at any time during growth specifies the exact quorum-sensing-controlled gene expression pattern (Lin *et al*., 2007; Pompeani *et al*., 2008; Rutherford *et al*., 2011).

Here we show that the Qrr sRNAs activate *aphA* through direct base pairing to its mRNA 5′ UTR. Activation is critical for high level production of AphA protein at low cell density, especially in *V. harveyi*, which exhibits a dramatic increase in AphA compared with that present at high cell density. Based on secondary structure predictions, the ∼ 200 nt long 5′ UTR of *aphA* mRNA is capable of forming an inhibitory structure masking its ribosome binding site, which presumably leads to translational inhibition. At low cell density, pairing of the Qrr sRNAs to the *aphA* mRNA 5′ UTR could disrupt this inhibitory structure and expose the ribosome binding site enabling AphA protein translation. Similar ‘anti-antisense’ mechanisms have been described for several other Hfq-chaperone-dependent *trans*-acting sRNAs including DsrA/RprA/ArcZ-*rpoS*, RyhB-*shiA* and GlmZ-*glmS* in *E. coli*, Qrr-*vca0939* in *V. cholerae* and recently, PhrS*-pqsR* in *Pseudomonas aeruginosa* (Majdalani *et al*., 1998; 2001; Hammer and Bassler, 2007; Prevost *et al*., 2007; Urban and Vogel, 2008; Mandin and Gottesman, 2010; Sonnleitner *et al*., 2011). We engineered 10 mutations (point mutations and deletions) in the *aphA* 5′ UTR in an attempt to disrupt the putative inhibitory structure and thereby increase basal AphA-GFP levels. None of these mutants exhibited increased GFP production (Fig. S6) indicating that multiple mutations in different regions of the *aphA* 5′ UTR are likely required to disrupt the inhibitory secondary structure.

What is the benefit of Qrr sRNA activation of *aphA*? Presumably during the transition from high cell density to low cell density, such as when vibrios exit a host or disperse from a biofilm, the immediate production of the Qrr sRNAs could promote rapid accumulation of AphA by both stabilizing and activating translation of *aphA* mRNA. This is especially noteworthy given that, in *V. harveyi*, AphA is undetectable at high cell density. Thus, a rapid and large fold change in AphA occurs at the high to low cell density transition. Presumably, going from ‘no’ AphA to a significant concentration of AphA enables a similar rapid and dramatic change in gene expression of AphA targets. We therefore propose that post-transcriptional rather than transcriptional activation of *aphA* could be crucial when an instantaneous switch in behavioural modes is required. Indeed, other such regulatory loops involving the Qrr sRNAs exist that affect quorum-sensing dynamics. LuxR/HapR activates *qrr* expression, which also increases the rapidity of the transition out of high cell density mode (Svenningsen *et al*., 2008; Tu *et al*., 2008). The Qrr sRNAs repress *luxO*, which delays the transition from low cell density to high cell density mode (Tu *et al*., 2010). Finally, the Qrr sRNAs repress *luxMN* encoding an AI-receptor pair, which adjusts the sensitivity of the quorum-sensing circuit to different AIs (Teng *et al*., 2011). Together, these loops exquisitely fine-tune the quorum-sensing transitions presumably to optimize survival in a changing environment. Moreover, we note that the Qrr sRNAs are used repeatedly in these various feedback loops, suggesting an economical solution to control quorum-sensing network dynamics.

As the universe of known bacterial sRNAs increases, two important themes are emerging: one is a scenario in which multiple sRNAs regulate the same target, for example, the sRNAs, DsrA, RprA and ArcZ all control the common target *rpoS*, which defines the gene expression pattern under different stress conditions (Majdalani *et al*., 1998; 2001; Mandin and Gottesman, 2010). The second scenario is one in which the same sRNA regulates multiple targets. For example, RyhB sRNA represses *sodB, iscS, cysE* and *fur* and it activates *shiA*, which together provide growth benefits under iron limiting conditions (Masse and Gottesman, 2002; Prevost *et al*., 2007; Vecerek *et al*., 2007; Desnoyers *et al*., 2009; Salvail *et al*., 2010), SgrS represses *ptsG* and *manX* to relieve sugar-phosphate stress (Vanderpool and Gottesman, 2004; Rice and Vanderpool, 2011), Spot42 controls genes in central and secondary metabolism (Moller *et al*., 2002a,b; Beisel and Storz, 2011), and RybB and MicA regulate genes encoding outer membrane proteins that counter cell envelope stress (Rasmussen *et al*., 2005; Udekwu *et al*., 2005; Johansen *et al*., 2006; Papenfort *et al*., 2006; Bossi and Figueroa-Bossi, 2007; Coornaert *et al*., 2010; Gogol *et al*., 2011). These many-to-one and one-to-many regulatory mechanisms give sRNAs overarching power in controlling regulatory networks. We frequently find multiple inputs are wired into sRNA production to ensure strict restriction of their levels, presumably to keep sRNA levels in check. The *V. harveyi* and *V. cholerae* Qrr sRNAs function by both scenarios: particular mRNA targets are regulated by multiple Qrr sRNAs and each Qrr sRNA controls multiple target mRNAs. Qrr sRNAs levels are precisely controlled through the feedback mechanisms described in the preceding paragraph. Furthermore, the level of each Qrr sRNA is affected by the other Qrr sRNAs due to dosage compensation (Svenningsen *et al*., 2009). Thus, coupling tight control of Qrr sRNA production to a large set of functions provides an orchestrated quorum-sensing response. Additional genes could be controlled by the Qrr sRNAs potentially providing links between quorum sensing and other regulatory networks.

Clearly, the Qrr sRNAs share overlapping functions; however, specificity is nonetheless ensured by several different means. First, in spite of their highly conserved sequences, there are particular regions of each Qrr sRNA that can be used to control distinct targets. As shown here, Qrr1 lacks one of the two pairing regions required for *aphA* activation, suggesting that Qrr1 prefers the targets *luxR* and *luxO*. Only about half of the nucleotides in the Qrr sRNAs are identical, suggesting that additional regions could exist to control other targets. It should in principle be possible to further separate regulation of *luxR* and *luxO* based on pairing differences. Indeed, mutating UGA (Figs 3A and 4A, mut iii) in Qrr4 has a more dramatic effect on *luxR* repression than on *luxO* repression (Fig. S5). At present, we only know a few Qrr targets, so this idea remains to be further explored as new Qrr targets are identified. Our findings are consistent with those for the sRNAs FnrS, GcvB and Spot42, which show that different stretches are used to control particular target mRNAs (Durand and Storz, 2010; Beisel and Storz, 2011; Sharma *et al*., 2011). Second, even when the pairing regions are conserved, differential regulation of target mRNAs could be achieved based on different expression levels and stabilities of the Qrr sRNAs. The contribution from each Qrr sRNA to regulation of each target mRNA will also be influenced by the efficacy of pairing and the stability of each Qrr-mRNA pair, which, in turn, depend on the avidity of their interactions with the Hfq chaperone and their secondary structures under particular physiological conditions (Vogel and Luisi, 2011). Third, differences in *qrr* promoter sequences suggest that each *qrr* is controlled by specific regulators. We know that phospho-LuxO regulates all the *qrr* genes; however, what additional environmental or intracellular cues affect the expression of one or a subset of the Qrr sRNAs remain undefined.

A key finding of this work is that in *Vibrionaceae* species possessing multiple *qrr* genes, Qrr1 lacks the region required for *aphA* activation. Species containing only *qrr*1 presumably reflect the ancestral state of this lineage. We suggest that duplication of the ancestral *qrr*1 gene in the lineage led to extant species containing multiple *qrr* genes. Region I in the Qrr sRNAs was co-opted for regulation of a new target, namely *aphA*. Subsequently, region I was lost from Qrr1, and the other Qrr sRNAs were relegated the role of controlling *aphA*. Because Qrr2–5 (*V. harveyi*) or Qrr2–4 (*V. cholerae*) contain redundant copies of region I, this region was most likely lost from Qrr1 as a consequence of neutral evolutionary drift. Loss of region I from Qrr1 in these species could be a neutral alteration to the quorum-sensing regulatory circuit. However, we suggest that there may be a selective advantage in possessing Qrr sRNAs devoted to particular regulatory roles, allowing finer tuning of the quorum-sensing circuit. If so, in species containing multiple Qrr sRNAs, Qrr1 could evolve the function of specific tuning of *luxR* and *luxO* expression.

The present work pinpoints a special role for Qrr1 in regulation of *aphA*; however, the other Qrr sRNAs could likewise have exclusive functions. Qrr5 is particularly interesting to us because it only exists in a subset of vibrios including *V. harveyi, Vibrio parahaemolyticus* and *Vibrio vulnificus* but not *V. cholerae* and *Vibrio splendidus*, which possess only Qrr1–4 (Lenz *et al*., 2004; Tu and Bassler, 2007; Miyashiro *et al*., 2010). Our previous studies show that, in *V. harveyi*, *qrr*5 is constitutively repressed under normal growth conditions (Tu and Bassler, 2007). However, Qrr5 is fully functional to repress *luxR*, *luxO*, and to activate *aphA* when expressed in *E. coli* (Tu and Bassler, 2007). Thus, it will be fascinating to learn under what conditions Qrr5 is produced in *V. harveyi*, and the functions of its specific target genes. In light of the above results, we predict that Qrr5 specific targets are conserved in vibrio species containing *qrr*5 but not in other vibrios.

## Experimental procedures

### Bacterial strains and growth conditions

*Vibrio harveyi* strain BB120 (BAA-1116) (Bassler *et al*., 1997) and derivatives were grown aerobically in Luria–Murine (LM) medium at 30°C. *V. cholerae* strain C6706 biovar El Tor (Thelin and Taylor, 1996) and derivatives were grown aerobically in Luria–Bertani (LB) medium at 30°C. *E. coli* strains S17-1λ*pir* and MC4100 were grown aerobically in LB medium at 37°C. Strains used in this study are described in Table S1. Antibiotics (Sigma) were used at the following concentrations: 200 µg ml^−1^ ampicillin (Amp), 100 µg ml^−1^ kanamycin (Kan), 10 µg ml^−1^ chloramphenicol (Cm), 100 µg ml^−1^ gentamicin (Gent), 10 µg ml^−1^ tetracycline (Tet), and 50 U ml^−1^ polymyxin B (Pb). *qrr* genes were induced with 10 mM rhamnose (Sigma). AphA-GFP and LuxO-GFP constructs were induced with 1 mM IPTG, while the LuxR-GFP construct was induced with 10 µM IPTG. Plasmid constructs were introduced into electrocompetent *E. coli* S17-1λ*pir* and MC4100 using 0.1 cm gap cuvettes (USA Scientific) and a Bio-Rad MicroPulser.

### DNA manipulations and mutant construction

*Escherichia coli* S17-1λ*pir* was used for all cloning procedures. DNA manipulations were performed as in (Sambrook *et al*., 1989). iProof DNA polymerase (Bio-Rad) was used for PCR reactions. Restriction enzymes, T4 DNA ligase, T4 polynucleotide kinase, and Antarctic phosphatase were purchased from New England Biolabs. Plasmids were constructed as described in Table S2 using primers listed in Table S3 from Integrated DNA Technologies (IDT). Site-directed mutagenesis was performed with the QuickChange II XL Site-Directed Mutagenesis Kit (Stratagene). All plasmids were confirmed by sequencing at Genewiz. Mutants in *V. harveyi* were constructed using λ red recombineering in *E. coli* S17-1λ*pir*::pKD46 (Datsenko and Wanner, 2000) on the pLAFR2 cosmid containing regions of the *V. harveyi* genome, followed by homologous recombination (Rutherford *et al*., 2011). *V. cholerae* mutants were constructed as described (Skorupski and Taylor, 1996).

### Western blot analysis

Cells at OD_600_ ∼ 1.0 were collected by centrifugation at 8000 *g* for 10 min and resuspended in TE buffer (10 mM Tris-HCl, pH 8.0 and 1 mM EDTA) with 0.5% SDS followed by sonication. Protein samples were analysed by SDS polyacrylamide gel electrophoresis (SDS-PAGE) on 12.5% gels, and wet transferred to nitrocellulose membranes at 100 volts for 1 h. Membranes were subsequently blocked in TBS-T with 5% milk for 1 h, incubated in primary antibody in TBS-T with 5% milk at a concentration of 1:3000 for 1 h, washed in TBS-T three times for 10 min each, incubated in secondary antibody in TBS-T with 5% milk at a concentration of 1:10 000 for 1 h, and again washed in TBS-T three times for 10 min each. Proteins were visualized using the Fast Western Blot Kit, ECL Substrate (Pierce). AphA antibody was generated in mice using purified AphA protein (Pocono Rabbit Farm & Laboratory). HRP conjugate anti-mouse IgG was used as the secondary antibody (Promega). Western blot results were quantified using ImageJ (Rasband, 1997–2011).

### RNA isolation and qRT-PCR

RNA used for quantitative RT-PCR (qRT-PCR) was isolated from *V. harveyi* and *V. cholerae* cultures at OD_600_ ∼ 1.0 using Trizol (Invitrogen) followed by DNase treatment (Ambion) and purification (Qiagen RNeasy) (Rutherford *et al*., 2011). cDNA was generated with SuperScript III reverse transcriptase (Invitrogen) using 1–3 µg of RNA. Real-time PCR analyses were performed on an ABI Prism 7900HT Sequence Detection System using Sybr Green mix (ABI). Triplicate biological samples were measured and analysed by a comparative CT method (Applied Biosystems) in which the relative amount of target RNA was normalized to the internal control RNA (*hfq*) first and subsequently to each other.

### GFP reporter assay

*Escherichia coli* strains were grown overnight aerobically at 37°C in LB medium with appropriate antibiotics, and diluted 1:1000 in triplicate into the identical medium containing the proper concentration of IPTG and rhamnose. GFP fluorescence and optical density OD_600_ were measured after 12–14 h of growth using an Envision 2103 Multilabel Reader (Perkin Elmer).
