# Supplementary material for: Quorum-sensing non-coding small RNAs use unique pairing regions to differentially control mRNA targets
Source: Mol Microbiol. 2012 Jan 9;83(3):599–611. doi: 10.1111/j.1365-2958.2011.07959.x (PMC3262071; doi:10.1111/j.1365-2958.2011.07959.x)
Supplement: Supplementary file 1 [file mmi0083-0599-SD1.pdf]

SUPPORTING INFORMATION

Figure S1 Qrr sRNAs activate *V. cholerae aphA* expression through base pairing
Figure S2 Qrr sRNA pairing predictions with *luxMN* and *vca0939*
Figure S3 Phylogenetic analyses of *aphA*, *luxR and luxO*
Figure S4 *aphA* sequence alignment
Figure S5 Qrr sRNAs use unique pairing regions to regulate different targets
Figure S6 Expression level of multiple AphA-GFP mutants in *E. coli*
Supplemental Table S1 Strains used in this study
Supplemental Table S2 Plasmids used in this study
Supplemental Table S3 Primers used in this study

A

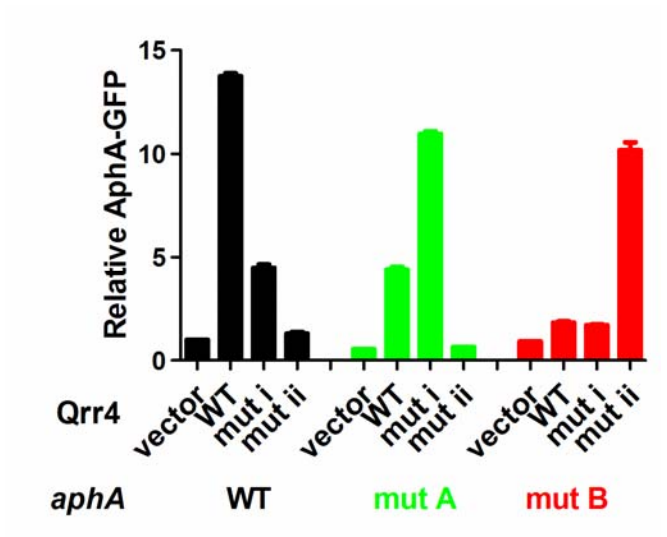

B

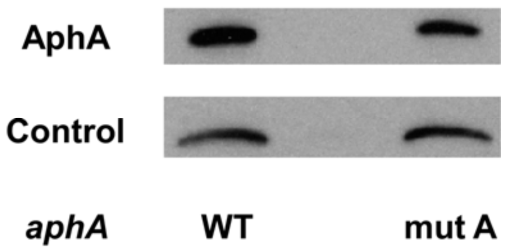

Figure S1 Qrr sRNAs activate *V. cholerae aphA* expression through base pairing

(A) Fluorescence from plasmid-encoded *V. cholerae* AphA-GFP (pYS143) or mutant AphA-GFP (pYS150, mutation A; pYS147, mutation B, denoted mut A and mut B as in Figure 3A) translational fusions were measured in *E. coli* MC4100 carrying an empty vector (pRHA109), a vector expressing a rhamnose-inducible *qrr*4 gene (pSTR0227) or a mutant *qrr*4 gene (pYS121, mutation I; pYS120, mutation II, denoted mut i and mut ii as in Figure 3A). GFP from three independent cultures was measured for each strain and the means and SEMs are shown. (B) AphA protein levels in a *V. cholerae ΔcqsA ΔluxQ* strain with wild type *aphA* (YZW477) or *aphA* carrying mutation A (see Figure 3A) (YS2013). Cells were harvested at OD_{600}~1.0, and protein levels were determined using Western blot.

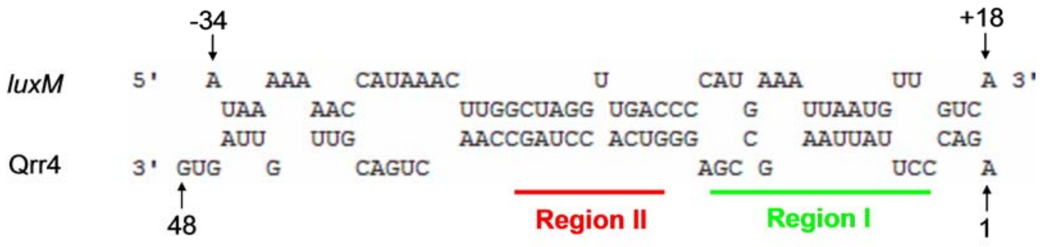

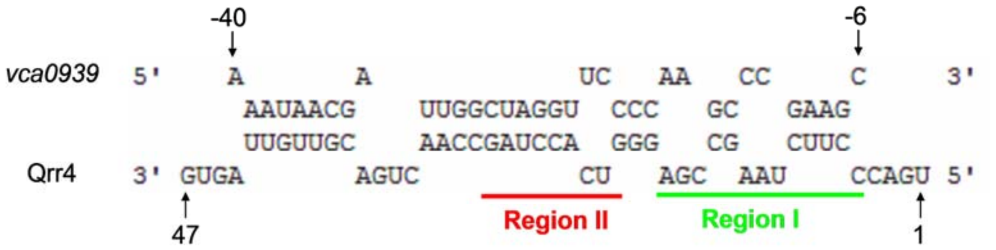

Figure S2 Qrr sRNA pairing predictions with *luxMN* and *vca0939*

Sequence alignment of *V. harveyi* Qrr4 (nucleotides1-48) with the *V. harveyi luxMN* mRNA and *V. cholerae* Qrr4 (nucleotides 1-47) with the *V. cholerae vca0939* mRNA, Region I and Region II are highlighted as in Figure 3A and 4A.

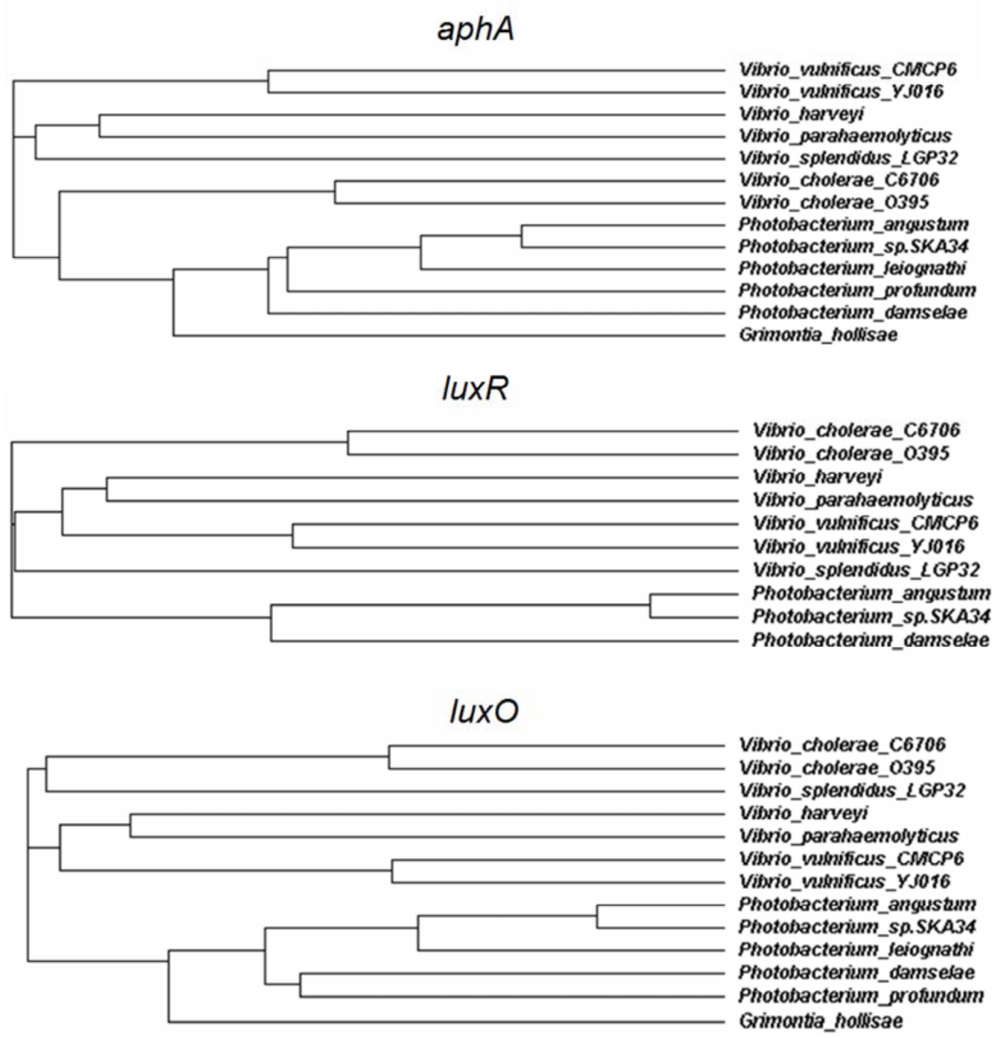

Figure S3 Phylogenetic analyses of *aphA*, *luxR and luxO*

Phylogenetic trees were built for *Vibrionaceae* species containing *aphA* genes.

```
Vibrio_cholerae_C6706      TATTCCACTTTATGCTTATTATTTAGATATACTACGTTCCCTCTGTGAT- 49
Vibrio_cholerae_O395       TATTCCACTTTATGCTTATTATTTAGATATACTACGTTCCCTCTGTGAT- 49
Vibrio_harveyi             TATTCCACTTCATGCTTATTATTTA--TATACTACTTCCCTGCTGGAAGC 48
Vibrio_parahaemolyticus    TATTCCACTTCATGCTTATTATTTA--TATACTACTTGCCTGCTGAGAGC 48
Vibrio_splendidus_LGP32    TATTCTACTTTATGCTTATTATTTAGATATACTACTGCTCCGCTGCAGA- 49
Vibrio_vulnificus_CMCP6    TATTCCACTTTATGCTTATTATTTAGATATACTACTCATCCGCTGCGGA- 49
Vibrio_vulnificus_YJ016    TATTCCACTTTATGCTTATTATTTAGATATACTACTCATCCGCTGCGGA- 49
                           ***** **** **************  ********    *  ***
```

Promoter **Transcription Start**

```
Vibrio_cholerae_C6706      AAGTAATGTAAAGCAATCTCACAGTTAAAGT--ATGCAAAGACATACGCC 97
Vibrio_cholerae_O395       AAGTAATGTAAAGCAATCTCACAGTTAAAGT--ATGCAAAGACATACGCC 97
Vibrio_harveyi             TCACAAATCCAATCAAATAAGCTCCAGCTC--GATGGAAAC-ATCCATCA 95
Vibrio_parahaemolyticus    TCATAAATCCAATCATACCTGCTCCAGCCCT-GATGGGGAT-CCCCATC- 95
Vibrio_splendidus_LGP32    -AACACTCCAAAATAATAAT-CTGCGGCACTCGACC-AATAAACTGGTCA 96
Vibrio_vulnificus_CMCP6    TCACACATCAAAATATTATTTCTGCAGC-CT-GATGGAATCGCTCCATCC 97
Vibrio_vulnificus_YJ016    TCACACATCAAA-TATTATTTCCGTAGC-CT-GATGGAATCACTCCATCC 96
                               *     **  *      *           *              *

Vibrio_cholerae_C6706      ACTCTAGGTGATAACCGGCTTTATAAGGTGACATAAGCAGC--CGAATTT 145
Vibrio_cholerae_O395       ACTCTAGGTGATAACCGGCTTTATAAGGTGACATAAGCAGC--CGAATTT 145
Vibrio_harveyi             ACTCTAGGTGATAAACGGCTTTA-A-GGTGACAGGACCAAC-ATTG--TT 140
Vibrio_parahaemolyticus    --TCTAGGTGATAAACGGCTTTA-A-GGTGACAGGACCAAC-TTTG--TT 138
Vibrio_splendidus_LGP32    A-CCTAGGTGATAAACGGCTTTGTAAGGTGACATAACCAACGATTGAGTT 145
Vibrio_vulnificus_CMCP6    ACCCTAGGTGATAAACGGCTTTA-AAGGTGACAGGACCAAC-ATTG--TT 143
Vibrio_vulnificus_YJ016    ACCCTAGGTGATAAACGGCTTTA-AAGGTGACAGGACCAAC-ATTG--TT 142
                              *********** *******  * *******  * ** *       **
```

Qrr Pairing Region

```
Vibrio_cholerae_C6706      TGCGCTGCAGGTATTTAAATGCGTTGATATGAGTGCCATTAGAAGCACAA 195
Vibrio_cholerae_O395       TGCGCTGCAGGTATTTAAATGCGTTGATATGCGTGCCATTAGAAGCACAA 195
Vibrio_harveyi             GGTGCTATCTACACATTAAAAAACTTA----AGCGTCATGAAGAACGCAA 186
Vibrio_parahaemolyticus    GGTGCTACTTATACATTAAAAAACTCA----AGCGCCAGTAAGAGCGCAG 184
Vibrio_splendidus_LGP32    GGTGCTACTTGAACATAACCAATATAA----CGTGCCA-TAGAAGCACAC 190
Vibrio_vulnificus_CMCP6    GGTGCTGACTGCACATAAACAAATT-------GCATCAACAAGAATGTAA 186
Vibrio_vulnificus_YJ016    GGTGCTGACTGCACATAAACAAATT-------GCATCAACAAGAATGTAA 185
                            * ***      *  * *      *       *   **  *  *    *

Vibrio_cholerae_C6706      CAACC-GTTTAGATAGAG-GTTTAT--GTTGACTTAATTTTTGGATTGAA 241
Vibrio_cholerae_O395       CAACC-GTTTAGATAGAA-GTTTAT--GTTGACTTAATTTTTGGATTGAA 241
Vibrio_harveyi             ATGAAAGTGTAGATAGCTTGTTTACAAGTTTATTGACCATTTGGATTGAA 236
Vibrio_parahaemolyticus    CTGAAAGTATAAGTAGCTTGTTTACAAGTTTATTGACCATTTGGATTGAA 234
Vibrio_splendidus_LGP32    TTGTT-GTTCAAGTA-TTTGTTTAC-----TGTTTACCATTTGGATTTCA 233
Vibrio_vulnificus_CMCP6    ACGCC-GTGCAGGCAGCTTGTTCATAAGTTTATTGACCATTTGGATTGAA 235
Vibrio_vulnificus_YJ016    ACGCC-GTGCAGGCAGCTTGTTCATAAGTTTATTGACCATTTGGATTGAA 234
                                 **   *   *    *** *         *  *   ********  *

Vibrio_cholerae_C6706      GACATGTCATTACCACACGTTATCCTTACTGTTCTTAGCACACGCGATGC 291
Vibrio_cholerae_O395       GACATGTCATTACCACACGTTATCCTTACTGTTCTTAGCACACGCGATGC 291
Vibrio_harveyi             GACATGTCATTACCACACGTAATTCTAACTGTACTTAGCACTCGCGACGC 286
Vibrio_parahaemolyticus    GACATGTCATTACCACACGTAATCTTAACTGTTCTTAGCACTCGCGACGC 284
Vibrio_splendidus_LGP32    GACATGTCATTACCACACGTAATTTTAACCGTTTTAAGTACACGCGATGC 283
Vibrio_vulnificus_CMCP6    GACATGTCATTACCACACGTAATTCTAACCGTTTTAAGCACTCGTGATGC 285
Vibrio_vulnificus_YJ016    GACATGTCATTACCACACGTAATTCTAACCGTTTTAAGCACTCGTGATGC 284
                           ******************** **  * ** **  * ** ** ** ** **
```

Start Codon

Figure S4 *aphA* sequence alignment

Sequence alignment of *aphA* genes in *Vibrionaceae* species with multiple Qrr sRNAs. Predicted promoters, conserved Qrr pairing regions, and start codons are indicated.

A

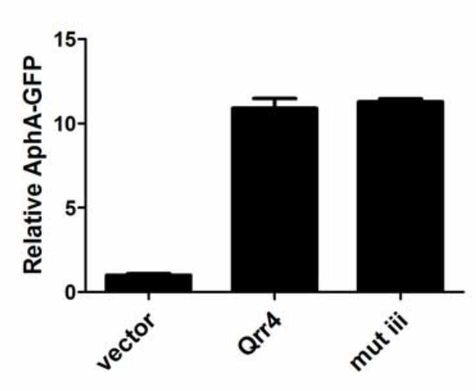

B

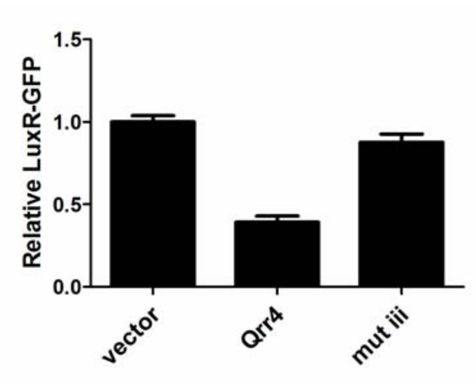

C

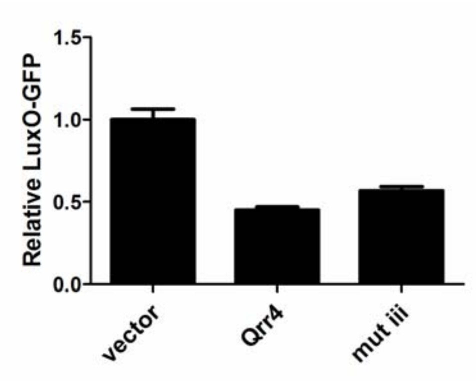

Figure S5 Qrr sRNAs use unique pairing regions to regulate different targets

(A)(B)(C) Fluorescence from plasmid-encoded *V. harveyi* AphA-GFP (pYS069), LuxR-GFP (pYS141) and LuxO-GFP (pYS142) translational fusions were measured in *E. coli* MC4100 carrying an empty vector (pRHA109), a vector expressing a rhamnose-inducible *qrr*4 gene (pSTR0227), or a mutant *qrr*4 gene (pYS153, denoted mut iii as in Figure 3A and 4A). GFP from three independent cultures was measured for each strain and the means and SEMs are shown.

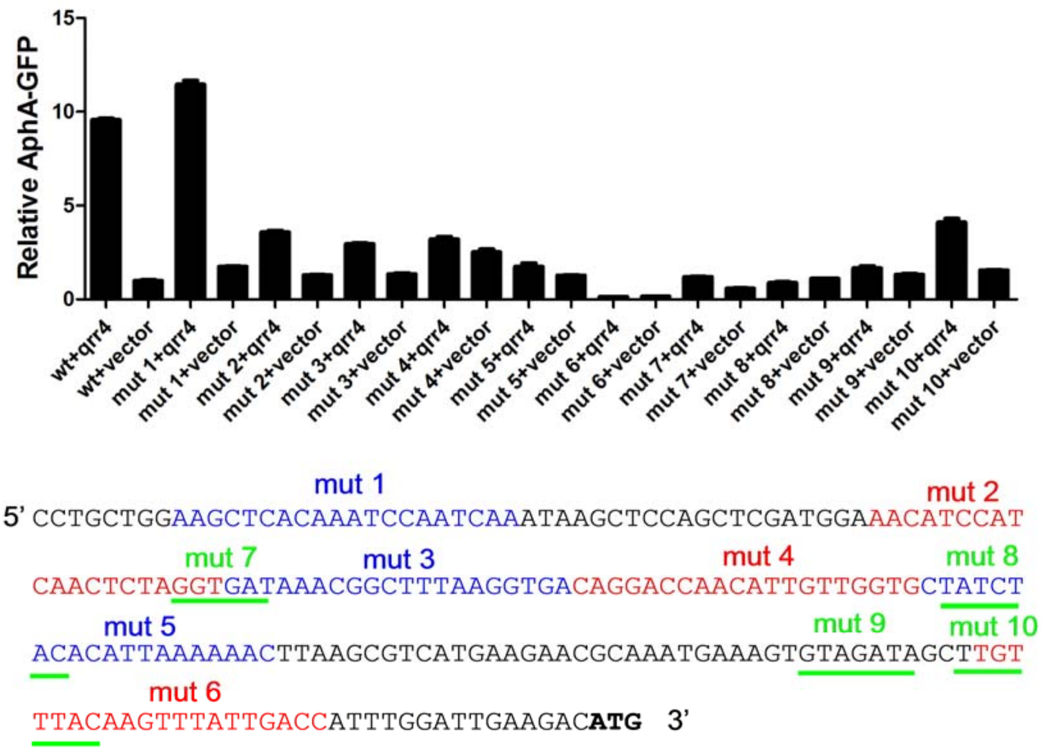

Figure S6 Expression level of multiple AphA-GFP mutants in *E. coli*

Fluorescence from a plasmid-encoded *V. harveyi* AphA-GFP translational fusion (pYS069, wt) was measured in *E. coli* MC4100 carrying an empty vector (pRHA109) or a vector expressing a rhamnose-inducible *qrr*4 gene (pSTR0227). Ten mutations in the *aphA* 5'UTR were engineered to disrupt the putative inhibitory structure. Fluorescence from these constructs was similarly measured. The mutations denoted mut 1 through mut 6 are deletions colored in blue and red. Mutations denoted mut 7 through mut 10 are point mutations colored in green. GFP from three independent cultures was measured for each strain and the means and SEMs are shown.

Supplemental Table S1. Strains used in this study.

Strain	Relevant Genotype	Source
E. coli		
S17λpir	wild type	(de Lorenzo & Timmis, 1994)
MC4100	wild type	(Casadaban, 1976)
V. harveyi		
BB120	wild type	(Bassler *et al.*, 1997)
KM83	*luxO*D47E	(Tu & Bassler, 2007)
KM669	*ΔluxR*	(Pompeani *et al.*, 2008)
KM812	*luxO*D47E *ΔluxR*	K. Mok, unpublished
YS040	*luxO*D47E *Δqrr*1-5 *ΔhapR*	this study
YS010	*ΔluxM ΔluxPQ ΔcqsS ΔluxR*	this study
YS034	YS010 with *aphA* mutation A	this study
V. cholerae		
C6706str2	wild type	(Thelin & Taylor, 1996)
SLS340	*luxO*D47E	S. Svenningsen, unpublished
SLS390	*ΔhapR*	(Svenningsen *et al.*, 2008)
SLS640	*luxO*D47E *ΔhapR*	S. Svenningsen, unpublished
SLS641	*luxO*D47E *Δqrr*1-4 *ΔhapR*	S. Svenningsen, unpublished
YZ477	*ΔcqsA ΔluxQ*	Y. Wei, unpublished
YS2013	YZ477 with *aphA* mutation A	this study

Supplemental Table S2. Plasmids used in this study.

Plasmid	Description	Source
pEVS143	empty vector	(Dunn *et al.*, 2006)
pYS069	pEVS143 with *V. harveyi* AphA-GFP	(Rutherford *et al.*, 2011)
pYS113	pYS069 with mutation A	this study
pYS112	pYS069 with mutation B	this study
pYS141	pEVS143 with *V. harveyi* LuxR-GFP	this study
pYS142	pEVS143 with *V. harveyi* LuxO-GFP	this study
pYS143	pEVS143 with *V. cholerae* AphA-GFP	this study
pYS150	pYS143 with mutation A	this study
pYS147	pYS143 with mutation B	this study
pRHA109	empty vector	(Giacalone *et al.*, 2006)
pYS122	pRHA109 with *V. harveyi qrr*1	this study
pSTR0227	pRHA109 with *V. harveyi qrr*4	(Rutherford et al., 2011)
pYS121	pSTR0227 with mutation i	this study
pYS120	pSTR0227 with mutation ii	this study
pYS153	pSTR0227 with mutation iii	this study
pLAFR2	empty vector	(Friedman *et al.*, 1982)

pBB39-3	pLAFR2 with *V. harveyi aphA*	(Bassler *et al.*, 1993)
pYS130	pBB39-3 with mutation A	this Study
pKAS32	empty vector	(Skorupski & Taylor, 1996)
pYS148	pKAS32 with *V. cholerae aphA*	this study
pYS152	pYS148 with mutation A	this study
pYS159	pLAFR2 with Δqrr1-luxOD47E	this study
pKD3	FRT sites flanking Cm^R gene template	(Datsenko & Wanner, 2000)
pKD46	Red recombinase expression plasmid	(Datsenko & Wanner, 2000)
pPH1JI	pLAFR2 incompatible plasmid	(Hirsch & Beringer, 1984)
pTL18	IPTG inducible FLP recombinase	(Long *et al.*, 2009)
pYS096	pYS069 with mutation 1	this study
pYS098	pYS069 with mutation 2	this study
pYS099	pYS069 with mutation 3	this study
pYS100	pYS069 with mutation 4	this study
pYS101	pYS069 with mutation 5	this study
pYS103	pYS069 with mutation 6	this study
pYS174	pYS069 with mutation 7	this study
pYS176	pYS069 with mutation 8	this study
pYS177	pYS069 with mutation 9	this study
pYS178	pYS069 with mutation 10	this study

Supplemental Table S3. Primers used in this study.

Primer	Sequence	Use
YS100	GCGGGTACCGCTAGCAAAGGAGAAGAACTC	pYS069/pYS141/pYS142/pYS143
YS101	GCGCCTAGGGTCGAGCTGTTTCCTGTGT	pYS069/pYS141/pYS142/pYS143
YS086	GCGCCTAGGCCTGCTGGAAGCTCACAAAT	pYS069
YS099	GCGGGTACCTACAGTTAGAATTACGTGTGGTAA	pYS069
YS335	ATCAACTCTAGGTGATAAACCCGATTAAGGTGACAGGACCAACA	pYS113
YS336	TGTTGGTCCTGTCACCTTAATCGGGTTTATCACCTAGAGTTGAT	pYS113
YS333	TGGAAACATCCATCAACTCTTCCAGATAAACGGCTTTAAGGTGA	pYS112
YS334	TCACCTTAAAGCCGTTTATCTGGAAGAGTTGATGGATGTTTCCA	pYS112
YS357	GCGCCTAGGCAATTAGGGGATTATCCCCAAAACATC	pYS141
YS358	GCGGGTACCAGTACGAGGTCTCTTTGCAATTGAG	pYS141
YS359	GCGCCTAGGAAAACACAACGAAAAATCGGCTAGGC	pYS142
YS360	GCGGGTACCAGACTTTTGACCTTCTGTTATTTGTTGCAT	pYS142
YS399	GCGCCTAGGCCTCTGTGATAAGTAATGTAAAGCAATC	pYS143
YS400	GCGGGTACCAACAGTAAGGATAACGTGTGGTAATGA	pYS143
YS443	CGCCACTCTAGGTGATAACCCCGATTATAAGGTGACATAAGCAG	pYS150/pYS152
YS444	CTGCTTATGTCACCTTATAATCGGGGTTATCACCTAGAGTGGCG	pYS150/pYS152
YS441	GCAAAGACATACGCCACTCTTCCAGATAACCGGCTTTATAAGGT	pYS147
YS442	ACCTTATAAAGCCGGTTATCTGGAAGAGTGGCGTATGTCTTTGC	pYS147
STR0067	CAGACGGTACCATATGCGGTGTG	pSTR0227/pYS122
STR0068	GTATCGTATACGACCAGTCTAAAAAGCG	pSTR0227/pYS122

STR0069	GGACCCCTCGGGTCACC	pYS122
STR0070	CAGTGGTACCTGCAGACAAAAAAGAA	pYS122
YS369	GGACCCCTCGGGTCACCTAGCCAACTGACGTTGTTAGTG	pYS122
YS370	CACTAACAACGTCAGTTGGCTAGGTGACCCGAGGGGTCC	pYS122
STR0075	AGACCCTTATTAAGCCGAGGGTCAC	pSTR0227
STR0076	CAGTGGTACCGCTCTAGAAAGAAAAAACGCCAATCACAATAAAGTTG	pSTR0227
YS351	CTGGTCGTAGACCCTTATTATCGGGAGGGTCACCTAGCCAACTG	pYS121
YS352	CAGTTGGCTAGGTGACCCTCCCGATAATAAGGGTCTACGACCAG	pYS121
YS349	CCCTTATTAAGCCGAGGGTCTGGAAGCCAACTGACGTTGTTAGT	pYS120
YS350	ACTAACAACGTCAGTTGGCTTCCAGACCCTCGGCTTAATAAGGG	pYS120
YS479	CCGAGGGTCACCTAGCCAACACTCGTTGTTAGTGAATACACAT	pYS153
YS480	ATGTGTATTCACTAACAACGAGTGTTGGCTAGGTGACCCTCGG	pYS153
YS377	CCTGCTGGAAGCTCACAAATC	pYS130
YS366	TACAGTTAGAATTACGTGTGGTAATGACAT	pYS130
YS367	ATGTCATTACCACACGTAATTCTAACTGTA	pYS130
YS290	GAGGATATTCATATGGACGAATTAGCCGATCACTTCAAG	pYS130
YS291	CATATGAATATCCTCCTTAGTTCCTATT	pYS130
YS292	TGTAGGCTGGAGCTGCTTCG	pYS130
YS293	CAGCTCCAGCCTACAACTTTAAATAAAACGAAAAAGGCTTGCCG	pYS130
YS376	CACTCTAGGTGTTCGTCCACAAG	pYS130
YS409	GCGGGTACCGCGCTACTTGAAGAGATGTGC	pYS148
YS412	GCGCCTAGGGCTGGAGTACCTGCTCGTATT	pYS148
YS451	GCGGGATCCGTAAAGAGACGCTGGTGGAGTTTG	pYS159
YS452	ATGCTGTATACTTTTATGCCCGAGCTATGGTCGTAG	pYS159
YS453	AAAAGTATACAGCATGGTTTGTGCC	pYS159
YS467	AGACGGAGCTCGAGCAGAATAAGATCAGGAATGCGATGGTTC	pYS159
YS468	TGCTCGAGCTCCGTCTACCTGATATGACGGGGATGGAC	pYS159
YS454	GAGGATATTCATATGCGTTGCGCTTAATGTCTTGCTCG	pYS159
YS455	CAGCTCCAGCCTACAACAAAGCCCTTCCGGTGTGGAA	pYS159
YS456	GCGGGATCCCTCGGAGCGTTTCGCGAACTG	pYS159
YS155	CTCGACCCTAGGCCTGCTGGATAAGCTCCAGCTCGATGGA	pYS096
YS156	TCCATCGAGCTGGAGCTTATCCAGCAGGCCTAGGGTCGAG	pYS096
YS159	ATAAGCTCCAGCTCGATGGAGATAAACGGCTTTAAGGTGA	pYS098
YS160	TCACCTTAAAGCCGTTTATCTCCATCGAGCTGGAGCTTAT	pYS098
YS161	AACATCCATCAACTCTAGGTCAGGACCAACATTGTTGGTG	pYS099
YS162	CACCAACAATGTTGGTCCTGACCTAGAGTTGATGGATGTT	pYS099
YS163	GATAAACGGCTTTAAGGTGACTATCTACACATTAAAAAAC	pYS100
YS164	GTTTTTTAATGTGTAGATAGTCACCTTAAAGCCGTTTATC	pYS100
YS165	CAGGACCAACATTGTTGGTGTTAAGCGTCATGAAGAACGC	pYS101
YS166	GCGTTCTTCATGACGCTTAACACCAACAATGTTGGTCCTG	pYS101
YS171	AAATGAAAGTGTAGATAGCTATTTGGATTGAAGACATGTC	pYS103
YS172	GACATGTCTTCAATCCAAATAGCTATCTACACTTTCATTT	pYS103
YS565	CTCGATGGAAACATCCATCAACTCTACCACTAAAACGGCTTTAAGGTGACAGGACCAA	pYS174
YS566	TTGGTCCTGTCACCTTAAAGCCGTTTTAGTGGTAGAGTTGATGGATGTTTCCATCGAG	pYS174

YS571	GGTGACAGGACCAACATTGTTGGTGCATAGATGACATTAAAAAACTTAAGCGTCATGAA	pYS176
YS572	TTCATGACGCTTAAGTTTTTTAATGTCATCTATGCACCAACAATGTTGGTCCTGTCACC	pYS176
YS573	GCGTCATGAAGAACGCAAATGAAAGTCATCTATGCTTGTTTACAAGTTTATTGACCATT	pYS177
YS574	AATGGTCAATAAACTTGTAAACAAGCATAGATGACTTTCATTTGCGTTCTTCATGACGC	pYS177
YS575	AGAACGCAAATGAAAGTGTAGATAGCAACAAATGAAGTTTATTGACCATTTGGATTGAAG	pYS178
YS576	CTTCAATCCAAATGGTCAATAAACTTCATTTGTTGCTATCTACACTTTCATTTGCGTTCT	pYS178

References

Bassler, B. L., E. P. Greenberg & A. M. Stevens, (1997) Cross-species induction of luminescence in the quorum-sensing bacterium Vibrio harveyi. *J Bacteriol* **179**: 4043-4045.

Bassler, B. L., M. Wright, R. E. Showalter & M. R. Silverman, (1993) Intercellular signalling in Vibrio harveyi: sequence and function of genes regulating expression of luminescence. *Mol Microbiol* **9**: 773-786.

Casadaban, M. J., (1976) Transposition and fusion of the lac genes to selected promoters in Escherichia coli using bacteriophage lambda and Mu. *J Mol Biol* **104**: 541-555.

Datsenko, K. A. & B. L. Wanner, (2000) One-step inactivation of chromosomal genes in Escherichia coli K-12 using PCR products. *Proc Natl Acad Sci U S A* **97**: 6640-6645.

de Lorenzo, V. & K. N. Timmis, (1994) Analysis and construction of stable phenotypes in gram-negative bacteria with Tn5- and Tn10-derived minitransposons. *Methods Enzymol* **235**: 386-405.

Dunn, A. K., D. S. Millikan, D. M. Adin, J. L. Bose & E. V. Stabb, (2006) New rfp- and pES213-derived tools for analyzing symbiotic Vibrio fischeri reveal patterns of infection and lux expression in situ. *Appl Environ Microbiol* **72**: 802-810.

Friedman, A. M., S. R. Long, S. E. Brown, W. J. Buikema & F. M. Ausubel, (1982) Construction of a broad host range cosmid cloning vector and its use in the genetic analysis of Rhizobium mutants. *Gene* **18**: 289-296.

Giacalone, M. J., A. M. Gentile, B. T. Lovitt, N. L. Berkley, C. W. Gunderson & M. W. Surber, (2006) Toxic protein expression in Escherichia coli using a rhamnose-based tightly regulated and tunable promoter system. *Biotechniques* **40**: 355-364.

Hirsch, P. R. & J. E. Beringer, (1984) A physical map of pPH1JI and pJB4JI. *Plasmid* **12**: 139-141.

Long, T., K. C. Tu, Y. Wang, P. Mehta, N. P. Ong, B. L. Bassler & N. S. Wingreen, (2009) Quantifying the integration of quorum-sensing signals with single-cell resolution. *PLoS Biol* **7**: e68.

Pompeani, A. J., J. J. Irgon, M. F. Berger, M. L. Bulyk, N. S. Wingreen & B. L. Bassler, (2008) The Vibrio harveyi master quorum-sensing regulator, LuxR, a TetR-type protein is both an activator and a repressor: DNA recognition and binding specificity at target promoters. *Mol Microbiol* **70**: 76-88.

Rutherford, S. T., J. C. van Kessel, Y. Shao & B. L. Bassler, (2011) AphA and LuxR/HapR reciprocally control quorum sensing in vibrios. *Genes Dev* **25**: 397-408.

Skorupski, K. & R. K. Taylor, (1996) Positive selection vectors for allelic exchange. *Gene* **169**: 47-52.

Svenningsen, S. L., C. M. Waters & B. L. Bassler, (2008) A negative feedback loop involving small RNAs accelerates Vibrio cholerae's transition out of quorum-sensing mode. *Genes Dev* **22**: 226-238.

Thelin, K. H. & R. K. Taylor, (1996) Toxin-coregulated pilus, but not mannose-sensitive hemagglutinin, is required for colonization by Vibrio cholerae O1 El Tor biotype and O139 strains. *Infect Immun* **64**: 2853-2856.

Tu, K. C. & B. L. Bassler, (2007) Multiple small RNAs act additively to integrate sensory information and control quorum sensing in Vibrio harveyi. *Genes Dev* **21**: 221-233.
